# Advances in E-Health

**DOI:** 10.3390/life11060468

**Published:** 2021-05-24

**Authors:** Vivian Vimarlund, Sabine Koch, Christian Nøhr

**Affiliations:** 1 Department of Computer and Information Science, School of Engineering, Linköping University, 581 83 Linköping, Sweden; vivian.vimarlund@liu.se; 2 Health Informatics Centre, Department of Learning, Informatics, Management and Ethics, Karolinska Institutet, 171 77 Stockholm, Sweden; sabine.koch@ki.se; 3 Center for Health Informatics and Technology, Maersk McKinney Moller Institute, University of Southern Denmark, 5230 Odense, Denmark

## 1. Introduction

E-health offers new ways to access health information, to deliver health and social care and to perform self-management. The adoption of e-health solutions by citizens, patients, health professionals, and healthcare providers has increased substantially during the last decade. For instance, in the European region, more than half of WHO member states have developed national e-health strategies [1]. A common goal in most strategies is to strengthen health systems through the implementation of digital technology. The World Health Organization has recently issued the Global Strategy on Digital Health (2020–2025) [2] to give guidance for the development and adoption of appropriate, accessible, affordable, scalable and sustainable person-centric digital health solutions to prevent, detect and respond to epidemics and pandemics, developing infrastructure and applications that enable countries to use health data to promote health and well-being, and to achieve the health-related Sustainable Development Goals.

To achieve this vision, evidence-based development of e-health services is crucial to offer high quality and effectiveness and to overcome some of the most challenging issues regarding accessibility, inequality, and equity in health and social care delivery, not least in times of pandemics.

The scope of this Special Issue is to outline some of the major challenges and future perspectives of e-health for health and social care delivery as well as self-management.

In this Special Issue, researchers share their latest achievements. It covers a diversity of work that reflects the state-of-the-art of issues and challenges regarding e-health design, implementation and adoption for a variety of stakeholders.

## 2. Presentation of the Papers

To provide an overview of available knowledge on the implementation and use of electronic health record (EHR) systems, including tethered personal health records (PHRs), the article by Tsai et al. [3] presents the current knowledge on the effects of EHR implementation and the barriers to EHR adoption and use. The authors identified positive as well as negative effects on the clinical work, data and information, patient care and economic impact. The most frequently reported barriers were resource constraints, insufficient training and support for users as well as poor literacy and skills in the technology. A difficulty for the accumulating knowledge in this area is the lack of uniformity in the use of EHR definitions and poor contextual information about the study settings.

In the light of the ongoing COVID-19 pandemic, many countries have experienced a dramatic increase in the use of digital technology to meet their doctor. This phenomenon has lead Demi et al. [4] to study the understanding of digital visits, as perceived by experts working in telemedicine companies. Nine managers and employees from eight telemedicine companies were interviewed, confirming that digital visits are important and efficient. Current digital visits cover approximately 30 to 35% of total consultations but the interviewed experts expected a dramatic rise should citizens make use of smart diagnostic devices that transmit data to physicians in real time.

Not only patient visits but also medical education has been affected by the COVID-19 pandemic, which is nicely illustrated by Franklin et al. During the lockdown of the University at Buffalo, 181 medical students completed their clinical training online by the use of telemedicine and tele-learning. The students were asked to answer questions that inquired about the students’ perspectives on their rapid switch from their traditional method of learning to the online version. They indicated that the top three specialties that were affected included surgery, internal medicine and obstetrics and gynecology. They were not so satisfied with the simulated cases in the learning material and would have preferred more ‘real’ cases. The medical students did not find tele-education and e-learning to be as effective as traditional medical education. However, it is concluded that telemedicine has a significant potential to address many of the challenges facing the medical education environment during pandemics such as the current COVID-19 pandemic [5].

We also experience how e-health technologies are increasingly being used to communicate information about medication. Monkman et al. [6] have reported a study exploring how information about prescription medications is best communicated to Canadian university students. In a time where several digital media are replacing paper-based communication, there is a need to know how the users of the information prefer to receive information about their prescription medicine to ensure they get the optimal benefits from the medicine, and that it is taken safely. The study was a qualitative investigation of young Canadian students’ preferences and rationale with respect to three different formats for receiving digital medication information: email, a mobile application and online. This particular user group preferred to receive the information by email followed by a mobile app. Other users may have different priorities, which suggest applying participatory methods among different user groups to explore their preferences. The flexibility of digital technologies enables multiple formats for communicating medication information.

Challenges about easy access, inequality, and equity in health and social care delivery are the issues for the article by Botin et al. [7]. The study focuses on citizens living with chronic conditions and how they are being marginalized or excluded from engagement despite recent developments in technology that otherwise aims to include. The paper argues that people-centered technology to support the chronic care systems must understand and respond to the needs and capacities of the most vulnerable in society. Furthermore, the paper suggests concrete approaches to move the healthcare system towards a more socially inclusive e-health system.

Whereas the previous studies focused on end-user perceptions of e-health applications, the paper by Wass and Safari [8] introduces a new design method to engage and empower participants with intellectual disability to participate in an innovation process. The findings in the study suggest that the particular method of applying photos as a part of the participatory process can enhance the sharing of contextual and individual needs. Furthermore, it was found that it also could increase empowerment including coping, self-determination and ownership.

The study by Chang et al. [9] complements the Special Issue with a technology perspective, offering a method to investigate relational factors for the adoption of cloud-based e-health systems based on multi-criteria decision-making. The intended level of adoption of an e-health cloud computing system could be determined by using the proposed approach. The results of a case study performed on the Taiwanese healthcare industry indicated that the cloud management function must be primarily enhanced, and that cost effectiveness is the most significant factor in the adoption of e-health cloud computing. This result is valuable for allocating resources to decrease performance gaps in the Taiwanese healthcare industry.

## 3. Discussion

The variety of issues studied in these Special Issue papers show how multifaceted e-health is as a scientific area, both in terms of focus but also in terms of methods applied. By contrast to this diversity, a summary of the findings from studies that have been conducted in North America, Europe and Asia show an increased interest in methods and tools to support patient-centric e-health development and vulnerable groups in order to increase inclusiveness, accessibility and equity. The papers give examples on how challenges to implementation and adoption might be overcome by adaptive solutions and stress the importance of high-quality study reporting regarding terminology and contextual information to allow for transferability of the results.
